# Pretransplant albumin-to-globulin ratio and long-term outcomes after liver transplantation for hepatocellular carcinoma: a two-center cohort study

**DOI:** 10.3389/fonc.2026.1885468

**Published:** 2026-08-24

**Authors:** Xun Liu, Xiao-An Liu, Di Wang, Jing Wang, Han-Xuan Wang, Ren Lang, Anshi Wu

**Affiliations:** 1 Anesthesia and Operation Center, Beijing Chao-Yang Hospital, Capital Medical University, Beijing, China; 2 Division of Hepatobiliary Surgery, Department of General Surgery, Beijing Chao-Yang Hospital, Capital Medical University, Beijing, China; 3 Department of Liver Transplantation, First Central Hospital of Tianjin Medical University, Tianjin, China; 4 Department of Thoracic Surgery, Beijing Chao-Yang Hospital, Capital Medical University, Beijing, China

**Keywords:** albumin-to-globulin ratio, competing risks, hepatocellular carcinoma, liver transplantation, overall survival, prognostic model, tumor recurrence

## Abstract

**Background:**

Prognosis after liver transplantation for hepatocellular carcinoma (HCC) is driven mainly by tumor burden, vascular invasion, and alpha-fetoprotein. Routine pretransplant nutritional indices may add information on recipient condition, but direct comparisons are scarce and recurrence is not always analyzed with competing mortality.

**Methods:**

The development cohort included 254 adults undergoing first liver transplantation for pathologically confirmed HCC. Overall survival (OS) was the primary outcome; time to recurrence (TTR) was analyzed with death before recurrence as a competing event. Five nutritional indices were compared using Kaplan-Meier estimates, Cox regression, and restricted cubic splines. AGR underwent subgroup and Fine-Gray analyses. Conventional staging, AGR alone, a pretransplant clinical model, and the clinical model plus AGR were compared by discrimination, calibration, and decision curves. Time-dependent ROC curves included bootstrap 95% confidence intervals. Fixed development-model parameters were evaluated in an independent external validation cohort of 58 recipients.

**Results:**

Eighty-five patients died and 51 experienced recurrence. In the fully adjusted Cox model, each 1-standard-deviation (SD) increase in AGR was associated with lower overall mortality [hazard ratio (HR), 0.64; 95% confidence interval (CI), 0.48-0.84; P = 0.002]; GNRI was also associated with OS (HR, 0.75; 95% CI, 0.58-0.98; P = 0.037). The AGR-OS association was nonlinear (overall P < 0.001; nonlinear P = 0.004). The adjusted recurrence subdistribution HR was 0.66 per 1-SD increase in AGR (95% CI, 0.47-0.94; P = 0.022). Adding AGR increased the OS C-index from 0.750 to 0.780 in the development cohort and from 0.645 to 0.686 externally. At 3 years, the external AUC was 0.713 (95% CI, 0.574-0.834) for the clinical model and 0.764 (95% CI, 0.614-0.888) after AGR was added.

**Conclusions:**

AGR showed the most consistent associations with OS and recurrence among the five indices. Its addition produced modest improvements in the development cohort, with changes in the same direction during external validation. AGR may complement, but should not replace, established tumor-related prognostic factors.

## 1 Introduction

Hepatocellular carcinoma (HCC) is the most common primary malignancy of the liver and usually arises in chronic liver disease, where persistent injury, fibrosis, and cirrhosis shape both treatment options and prognosis (1–3). Clinically useful risk assessment must therefore account for tumor behavior as well as the condition of the host liver.

Liver transplantation addresses both components by removing the tumor-bearing liver and the underlying diseased organ. Outcomes nonetheless vary after transplantation. Recurrence, vascular invasion, alpha-fetoprotein (AFP), pathological tumor burden, perioperative complications, and post-transplant immunosuppression all contribute to subsequent risk (4–8). Blood-based indices cannot characterize the tumor microenvironment directly, but they can provide a practical summary of the recipient’s systemic and hepatic condition before transplantation.

Several indices derived from routine laboratory and anthropometric measurements have been used for nutritional or prognostic assessment. CONUT combines albumin, lymphocyte count, and cholesterol; PNI combines albumin and lymphocyte count; GNRI combines albumin with the ratio of actual to ideal body weight; TCBI incorporates triglycerides, cholesterol, and body weight; and AGR relates albumin to globulin (9–14). These measures emphasize different aspects of hepatic synthesis, nutritional reserve, lipid metabolism, and systemic illness. Their performance has rarely been compared within the same HCC transplant cohort, particularly with recurrence analyzed as a competing-risk outcome.

We therefore compared the five indices under a common analytic framework. OS was retained as the primary endpoint, and TTR was examined as a clinically focused secondary endpoint with death before recurrence treated as a competing event. AGR underwent further exploratory analyses after showing the most consistent pattern in the development cohort. Its incremental prognostic contribution was assessed alongside established tumor-related variables and then examined in an independent external validation cohort.

## 2 Methods

### 2.1 Study design and participants

This retrospective observational study included consecutive adults aged 18 years or older who underwent first liver transplantation at Beijing Chao-Yang Hospital, Capital Medical University, between April 28, 2013 and June 30, 2023, and had HCC confirmed in the explanted liver. Follow-up closed on July 1, 2024. Patients were required to have perioperative records and the pretransplant laboratory and anthropometric measurements needed to calculate all five indices. Exclusion criteria were combined hepatocellular-cholangiocarcinoma, another concurrent malignancy, multiorgan or living-donor transplantation, and missing measurements required for index calculation. All eligible patients during the study period were included; no formal sample-size calculation was undertaken.

An independent external validation cohort was assembled at the Department of Liver Transplantation, First Central Hospital of Tianjin Medical University, Tianjin, China. It comprised 58 adults who underwent liver transplantation for HCC in 2018 and were followed through December 31, 2023. Median follow-up calculated by the reverse Kaplan-Meier method was 65.5 months, and the maximum observed follow-up was 71.8 months. Recipients were managed at a separate institution by an independent clinical and surgical team. Eligibility criteria, baseline variable definitions, and outcome coding were harmonized with those of the development cohort before model evaluation.

### 2.2 Data collection and data quality

Demographic variables included age, sex, height, weight, and body mass index (BMI). Tumor variables comprised number and maximum diameter on pretransplant imaging, portal vein tumor thrombus (PVTT), AFP, capsule invasion, microvascular invasion (MVI), AJCC T stage, Barcelona Clinic Liver Cancer (BCLC) stage, China Liver Cancer (CNLC) stage, and Milan-criteria status. Liver-related variables included Child-Pugh class, Model for End-Stage Liver Disease (MELD) score, cirrhosis, portal hypertension, and pretransplant ascites. Laboratory measurements included albumin, globulin, total protein, total cholesterol, triglycerides, lymphocyte count, and platelet count. The initial immunosuppressive regimen and use of calcineurin inhibitors (CNIs), mycophenolate mofetil (MMF), and mechanistic-target-of-rapamycin (mTOR) inhibitors were recorded when available.

For each index, the blood sample closest to transplantation and obtained within the preceding 7 days was used. Laboratory units were harmonized before calculation. Survival and recurrence times were required to be nonnegative, and recurrence times after death or final follow-up were flagged during quality control. AGR was recalculated from source albumin and globulin values. Records with nonpositive globulin or nonfinite AGR would have been excluded, but none were present. No statistical imputation was used for model covariates, and models were fitted to their stated complete-case populations. Cohort-wide occurrence data for infection within 90 days and acute rejection within 1 year were unavailable in the development cohort and were retained as missing rather than coded as no event. Infection-related and rejection-related deaths were separate cause-of-death classifications and were not used to infer complication incidence. The same range and consistency checks were applied to the external validation data.

### 2.3 Nutritional indices and grouping criteria

The five indices were calculated using the formulas in **Table 1**. CONUT scoring followed the published albumin, lymphocyte, and cholesterol criteria; for analysis, the original mild and moderate categories were combined, giving groups of 0-1, 2-8, and 9–12 points (9). PNI was calculated as albumin (g/L) plus five times the lymphocyte count (10^9/L) (10). GNRI was calculated as 1.489 times albumin (g/L) plus 41.7 times the ratio of actual to ideal body weight, with the weight ratio capped at 1; the original low- and moderate-risk groups were combined to form categories of <82, 82-98, and >98 (11). TCBI was triglycerides (mg/dL) multiplied by total cholesterol (mg/dL) and body weight (kg), divided by 1,000 (12). AGR was albumin divided by globulin, with both expressed in g/L (13). TCBI was included because its lipid- and weight-based construction differs from the albumin-centered indices.

**Table 1:** Calculation formulas and grouping criteria for pretransplant nutritional indices.

Index	Formula	Grouping criteria
CONUT	Albumin score + lymphocyte score + total cholesterol score. Albumin (g/dL): >=3.5 = 0, 3.0-3.49 = 2, 2.5-2.99 = 4, <2.5 = 6; lymphocytes (/mm3): >=1600 = 0, 1200-1599 = 1, 800-1199 = 2, <800 = 3; cholesterol (mg/dL): >=180 = 0, 140-179 = 1, 100-139 = 2, <100 = 3.	Normal 0-1; mild-to-moderate malnutrition 2-8; severe malnutrition 9-12.
GNRI	1.489 x albumin (g/L) + 41.7 x (actual weight/ideal weight); weight ratio capped at 1. Ideal weight: male = height - 100 - (height - 150)/4; female = height - 100 - (height - 150)/2.5.	High risk <82; borderline-to-moderate risk 82-98; normal >98.
PNI	Albumin (g/L) + 5 x total lymphocyte count (10^9/L).	Development-cohort tertiles: T1 <= 36.72; T2 > 36.72 to <= 43.00; T3 > 43.00.
TCBI	Triglycerides (mg/dL) x total cholesterol (mg/dL) x body weight (kg)/1000. mmol/L converted to mg/dL before calculation.	Development-cohort tertiles: T1 <= 597.70; T2 > 597.70 to <= 1143.68; T3 > 1143.68.
AGR	Albumin (g/L)/globulin (g/L).	Development-cohort tertiles: T1 <= 1.0370; T2 > 1.0370 to <= 1.3871; T3 > 1.3871.

AGR, albumin-to-globulin ratio; CONUT, controlling nutritional status score; GNRI, geriatric nutritional risk index; PNI, prognostic nutritional index; TCBI, triglyceride-total cholesterol-body weight index. PNI, TCBI, and AGR cut points were derived in the development cohort and fixed for the independent external validation cohort.

PNI, TCBI, and AGR were categorized by development-cohort tertiles to preserve the original comparative framework. The cut points were 36.72 and 43.00 for PNI, 597.70 and 1,143.68 for TCBI, and 1.0370 and 1.3871 for AGR. These values were used for every grouped analysis and were not recalculated in the external validation cohort. Continuous analyses were primary for regression because they retain more information; each index was standardized using the development-cohort mean and SD.

### 2.4 Outcomes and follow-up

OS was defined from transplantation to death from any cause; recipients alive at last contact were censored on that date. Cause of death was summarized as recurrence-related, infection-related, rejection-related, other, or unclassified. These categories were descriptive and were not used to fit cause-specific survival models.

TTR was measured from transplantation to the first documented HCC recurrence. Follow-up ended at recurrence, death without previous recurrence, or last contact. Recurrence was coded as the event of interest, death before recurrence as the competing event, and event-free follow-up as censored. Recurrence probability was estimated from the cumulative incidence function rather than from one minus the Kaplan-Meier estimator.

### 2.5 Descriptive analysis

Continuous variables are summarized as mean and SD and were compared between patients alive and deceased at the end of follow-up using the Student t test when appropriate. Categorical variables are reported as number and percentage and were compared using the chi-square test or Fisher exact test. Missing counts are reported for incomplete variables. Baseline P values were descriptive and were not used to select covariates for multivariable models.

### 2.6 Overall-survival analysis

Kaplan-Meier estimates and log-rank tests compared OS across the prespecified categories of each index. Cox proportional-hazards models were fitted for standardized continuous indices and for grouped indices. Model 1 was unadjusted. Model 2 included age and sex. Model 3 included age, sex, ln(AFP), maximum tumor diameter, tumor number, PVTT, and Child-Pugh class. These covariates were selected on clinical grounds to represent demographics, tumor biology and burden, macrovascular invasion, and hepatic reserve while limiting model complexity relative to the 85 observed deaths. Composite staging systems were not entered with their component variables.

Hazard ratios (HRs) are reported with 95% confidence intervals (CIs). The proportional-hazards assumption for the focused AGR model was examined using scaled Schoenfeld residuals for individual terms and the global model. No departure was detected for AGR (P = 0.599) or globally (P = 0.804). Statistical inference for the five-index comparison was based on the complete 254-patient development cohort.

### 2.7 Dose-response and subgroup analyses

Restricted cubic splines with four knots were fitted for each continuous index in Model 3. The median value was used as the reference, and Wald tests assessed the overall association and departure from linearity. Curves were restricted to the displayed observed range and were used to examine dose-response shape, not to derive new cut points.

AGR subgroup analyses were performed by age (<60 or ≥60 years), sex, BMI (<24 or ≥24 kg/m²), Child-Pugh class (A or B/C), Milan criteria, PVTT, tumor number (1 or >1), and maximum tumor diameter (≤5 or >5 cm). Models used the Model 3 covariates except for the variable defining the stratum. Heterogeneity was assessed with multiplicative interaction terms and nested-model comparisons. Because the initial review raised a Child-Pugh interaction, we also fitted a three-level AGR-by-Child-Pugh interaction and estimated class-specific associations for classes A, B, and C. Subgroup analyses were exploratory.

### 2.8 Recurrence and competing-risk regression

Cumulative recurrence incidence across AGR tertiles was compared using Gray’s test (15). Fine-Gray subdistribution-hazards regression was used to estimate the association between AGR and recurrence while retaining deaths without recurrence in the risk sets (16). Adjustment followed the OS sequence: Model 1 was unadjusted; Model 2 included age and sex; and Model 3 additionally included ln(AFP), maximum tumor diameter, tumor number, PVTT, and Child-Pugh class. AGR was evaluated per 1-SD increase and by tertile. Results are reported as subdistribution HRs (sHRs) with 95% CIs. Cause-specific Cox regression was not performed because the analysis addressed cumulative recurrence probability in the presence of competing mortality.

### 2.9 Prediction models, calibration, and clinical utility

Five OS models were evaluated in a common complete-case sample: BCLC stage, CNLC stage, AGR alone, a pretransplant clinical Cox model, and the clinical model plus continuous AGR. The clinical model included age, sex, ln(AFP), maximum tumor diameter, tumor number, PVTT, and Child-Pugh class. The direct ROC comparison was restricted to AGR alone, the clinical model, and the clinical-plus-AGR model. TTR prediction compared Milan status, a clinical Fine-Gray model, and the same model plus AGR. Staging systems were evaluated separately because they incorporate tumor features also represented individually in the clinical models.

OS model performance was summarized by Harrell’s C-index and the Akaike information criterion. Time-dependent AUCs and Brier scores were estimated at 1, 3, and 5 years, with 200 bootstrap cross-validation resamples for internal validation. Cumulative/dynamic ROC curves were constructed from fixed model prediction scores for AGR alone, the clinical model, and the clinical-plus-AGR model. Pointwise curve bands and AUC confidence intervals were obtained from 500 patient-level nonparametric bootstrap samples within each evaluation cohort. The 3-year horizon was emphasized in the main analysis; 1- and 5-year curves were retained in the **Supplementary Material**. Three-year calibration compared grouped mean predicted risks with observed risks. Decision curves used jackknife pseudo-observations to estimate net benefit across clinically plausible thresholds (17, 18).

### 2.10 Exploratory machine-learning analysis

The exploratory machine-learning analysis compared candidate survival models, including penalized Cox regression, stepwise Cox regression, gradient boosting, and random survival forests (19). The data were divided into training and testing sets using a fixed random seed, and discrimination was summarized by the training, testing, and mean Harrell C-indices. The model with the highest mean C-index was selected for further interpretation.

For the selected random survival forest, variable importance and SHapley Additive exPlanations (SHAP) were used to describe the contribution of individual variables to the fitted risk score (20). This analysis was exploratory and was not used to define the primary endpoint, select covariates for the clinical Cox model, or determine the AGR-focused analyses.

### 2.11 Immunosuppression and external validation

Initial immunosuppressive regimens and CNI class were summarized descriptively. MMF and mTOR-inhibitor use were derived from the recorded initial regimen. Missing fields for 90-day infection and 1-year acute rejection were not recoded as absence. These post-transplant events and later treatment changes were not included in the primary model because they occurred after measurement of the pretransplant exposure and could lie on the pathway to outcome. A sensitivity Cox model added initial CNI class to the pretransplant clinical model.

For external validation, the development-cohort coefficients, baseline hazards, standardization parameters, and AGR cut points were fixed and applied to the independent external validation cohort without refitting. Harrell’s C-index, time-dependent AUCs with bootstrap 95% confidence intervals, Brier scores, and calibration were calculated for OS at 1, 3, and 5 years. The ROC analysis compared AGR alone, the clinical model, and the clinical-plus-AGR model using the transported development-model prediction scores. Calibration slopes and grouped observed-versus-predicted risks were examined at 3 and 5 years, when adequate follow-up remained. BCLC and CNLC were retained as conventional staging benchmarks in the development cohort; external transport focused on the fixed clinical and clinical-plus-AGR models. External TTR discrimination was secondary because only 12 recurrences occurred.

### 2.12 Statistical software, multiplicity, and ethics

Analyses were performed in R version 4.4.3. All tests were two-sided, with P < 0.05 denoting statistical significance. Comparisons across five indices, alternative scales, splines, and subgroup analyses were exploratory; P values were nominal and were not adjusted for multiplicity. Interpretation considered effect size, CI width, consistency across analyses, and prediction performance rather than statistical significance alone.

The Ethics Review Committee of Beijing Chao-Yang Hospital, Capital Medical University, approved the development study (2026-Ke-528) and waived written informed consent for this retrospective analysis of de-identified data. The external validation study was approved by the Ethics Committee of First Central Hospital of Tianjin Medical University (approval no. 2020N252KY). The external validation dataset was de-identified before transfer and analysis. The study was conducted in accordance with the Declaration of Helsinki and institutional requirements. Liver grafts were obtained through the official Chinese donation and allocation system with the required consent and oversight; no grafts were obtained from prisoners or other nonconsenting sources.

## 3 Results

### 3.1 Cohort and baseline characteristics

The development cohort contained 254 recipients, of whom 169 (66.5%) were alive and 85 (33.5%) had died by the last follow-up. Median follow-up was 67.0 months. The mean age was 57.48 ± 10.10 years, 211 patients (83.1%) were men, and the mean BMI was 24.80 ± 3.48 kg/m². Age, sex, height, weight, and BMI did not differ between survivors and decedents (all P>0.05). All five nutritional indices could be calculated in every recipient.

The deceased group had a greater tumor burden and more aggressive pathological features. Maximum tumor diameter was larger than in the alive group (6.09 ± 4.23 vs. 3.50 ± 2.61 cm; P < 0.001), and the distribution of tumor number differed (P < 0.001). PVTT was present in 28.2% of deceased recipients and 7.1% of those alive, capsule invasion in 55.3% and 24.9%, and MVI in 83.5% and 42.0%, respectively (all P < 0.001). Mean ln(AFP) was also higher in the deceased group (5.15 ± 2.71 vs. 3.48 ± 2.03; P < 0.001), and AJCC T-stage distributions differed between groups (P < 0.001).

CNLC stage and Milan-criteria status also differed by survival status. Among recipients with recorded CNLC stage, stage 3A accounted for 20.7% of the deceased group and 5.1% of the alive group (overall P < 0.001). The proportions within Milan criteria were 21.2% and 59.2%, respectively (P < 0.001). BCLC stage did not differ significantly (P = 0.300). No between-group difference was detected for Child-Pugh class, MELD score, pretransplant ascites, portal hypertension, or recorded cirrhosis.

Albumin was lower in the deceased group than in the alive group (33.91 ± 4.19 vs. 35.45 ± 6.33 g/L; P = 0.022), whereas globulin was higher (32.61 ± 7.23 vs. 28.61 ± 8.15 g/L; P < 0.001). Platelet count was also higher (119.80 ± 73.84 vs. 91.38 ± 57.44 × 10^9/L; P = 0.002). Total protein, triglycerides, and lymphocyte count did not differ. GNRI was lower among deceased recipients (91.27 ± 7.12 vs. 93.87 ± 9.28; P = 0.014), and AGR was 1.10 ± 0.30 versus 1.36 ± 0.49 (P < 0.001). CONUT, PNI, and TCBI showed no significant between-group differences. Complete baseline data are reported in **Table 2**.

**Table 2:** Baseline characteristics according to overall survival status.

Variable	Overall (N=254)	Survival (n=169)	Death (n=85)	P value
Age (years)	57.48 ± 10.10	57.46 ± 9.68	57.51 ± 10.96	>0.900
Sex				0.400
Female	43 (16.9%)	31 (18.3%)	12 (14.1%)	
Male	211 (83.1%)	138 (81.7%)	73 (85.9%)	
Height (cm)	170.50 ± 6.61	170.27 ± 6.52	170.98 ± 6.81	0.400
Weight (kg)	72.22 ± 11.60	72.35 ± 11.08	71.98 ± 12.64	0.800
BMI (kg/m2)	24.80 ± 3.48	24.93 ± 3.41	24.56 ± 3.63	0.400
Tumor number	Overall (N=254)	Survival (n=169)	Death (n=85)	<0.001
1	118 (46.5%)	87 (51.5%)	31 (36.5%)	
2	49 (19.3%)	42 (24.9%)	7 (8.2%)	
3	42 (16.5%)	24 (14.2%)	18 (21.2%)	
4	16 (6.3%)	5 (3.0%)	11 (12.9%)	
5	22 (8.7%)	9 (5.3%)	13 (15.3%)	
6	5 (2.0%)	1 (0.6%)	4 (4.7%)	
7	2 (0.8%)	1 (0.6%)	1 (1.2%)	
Portal vein tumor thrombus				<0.001
No	218 (85.8%)	157 (92.9%)	61 (71.8%)	
Yes	36 (14.2%)	12 (7.1%)	24 (28.2%)	
ln (AFP)	4.04 ± 2.41	3.48 ± 2.03	5.15 ± 2.71	<0.001
Capsule invasion				<0.001
No	165 (65.0%)	127 (75.1%)	38 (44.7%)	
Yes	89 (35.0%)	42 (24.9%)	47 (55.3%)	
Microvascular invasion				<0.001
No	112 (44.1%)	98 (58.0%)	14 (16.5%)	
Yes	142 (55.9%)	71 (42.0%)	71 (83.5%)	
AJCC T stage				<0.001
1	65 (25.6%)	61 (36.1%)	4 (4.7%)	
1A	2 (0.8%)	2 (1.2%)	0 (0.0%)	
1B	2 (0.8%)	2 (1.2%)	0 (0.0%)	
2	133 (52.4%)	86 (50.9%)	47 (55.3%)	
3	37 (14.6%)	16 (9.5%)	21 (24.7%)	
4	15 (5.9%)	2 (1.2%)	13 (15.3%)	
BCLC stage				0.300
A	94 (37.0%)	68 (40.2%)	26 (30.6%)	
B	108 (42.5%)	67 (39.6%)	41 (48.2%)	
D	52 (20.5%)	34 (20.1%)	18 (21.2%)	
CNLC stage				<0.001
1A	54 (22.7%)	47 (30.1%)	7 (8.5%)	
1B	68 (28.6%)	45 (28.8%)	23 (28.0%)	
2A	21 (8.8%)	15 (9.6%)	6 (7.3%)	
2B	18 (7.6%)	7 (4.5%)	11 (13.4%)	
3A	25 (10.5%)	8 (5.1%)	17 (20.7%)	
4	52 (21.8%)	34 (21.8%)	18 (22.0%)	
Missing	16	13	3	
Within Milan criteria				<0.001
Outside	136 (53.5%)	69 (40.8%)	67 (78.8%)	
Within	118 (46.5%)	100 (59.2%)	18 (21.2%)	
Child-Pugh class				0.300
A	81 (31.9%)	50 (29.6%)	31 (36.5%)	
B	116 (45.7%)	83 (49.1%)	33 (38.8%)	
C	57 (22.4%)	36 (21.3%)	21 (24.7%)	
MELD score	12.42 ± 5.93	12.70 ± 6.25	11.88 ± 5.27	0.300
Missing	16	13	3	
Cirrhosis				>0.900
No	6 (3.0%)	4 (2.9%)	2 (3.2%)	
Yes	192 (97.0%)	132 (97.1%)	60 (96.8%)	
Missing	56	33	23	
Portal hypertension				0.800
No	86 (33.9%)	58 (34.3%)	28 (32.9%)	
Yes	168 (66.1%)	111 (65.7%)	57 (67.1%)	
Preoperative ascites				>0.900
No	161 (63.4%)	107 (63.3%)	54 (63.5%)	
Yes	93 (36.6%)	62 (36.7%)	31 (36.5%)	
Albumin (g/L)	34.93 ± 5.74	35.45 ± 6.33	33.91 ± 4.19	0.022
Globulin (g/L)	29.95 ± 8.06	28.61 ± 8.15	32.61 ± 7.23	<0.001
Total protein (g/L)	64.77 ± 9.33	64.06 ± 9.50	66.18 ± 8.88	0.082
Cholesterol (mmol/L)	3.52 ± 1.30	3.36 ± 1.34	3.84 ± 1.14	0.003
Triglycerides (mmol/L)	1.22 ± 1.04	1.22 ± 1.07	1.22 ± 1.01	>0.900
Lymphocyte count (10^9/L)	1.02 ± 0.66	1.02 ± 0.68	1.02 ± 0.62	>0.900
Platelet count (10^9/L)	100.89 ± 64.67	91.38 ± 57.44	119.80 ± 73.84	0.002
CONUT	5.04 ± 2.84	5.21 ± 2.92	4.69 ± 2.65	0.200
GNRI	93.00 ± 8.70	93.87 ± 9.28	91.27 ± 7.12	0.014
PNI	40.02 ± 7.63	40.54 ± 8.26	38.99 ± 6.12	0.093
TCBI	1,142.13 ± 1,321.50	1,124.10 ± 1,492.49	1,177.98 ± 896.03	0.700
AGR	1.27 ± 0.45	1.36 ± 0.49	1.10 ± 0.30	<0.001
Initial CNI class				0.300
CSA-based	51 (20.1%)	38 (22.5%)	13 (15.3%)	
TAC-based	198 (78.0%)	127 (75.1%)	71 (83.5%)	
TAC+CSA combined/switched	5 (2.0%)	4 (2.4%)	1 (1.2%)	

Continuous variables are presented as mean ± SD and categorical variables as n (%). P values are shown to three decimal places. AFP, alpha-fetoprotein; AGR, albumin-to-globulin ratio; BMI, body mass index; CNI, calcineurin inhibitor; MVI, microvascular invasion; PVTT, portal vein tumor thrombus.

### 3.2 Cox regression analyses

Cox regression results are shown in **Table 3**. For continuous AGR, the HR per 1-SD increase was 0.61 in Model 1 (95% CI, 0.47-0.78; P < 0.001), 0.61 in Model 2 (95% CI, 0.47-0.79; P < 0.001), and 0.64 in Model 3 (95% CI, 0.48-0.84; P = 0.002). The proportional-hazards tests were nonsignificant for AGR (P = 0.599) and for the global Model 3 test (P = 0.804). In the fully adjusted categorical analysis, mortality was lower for AGR T3 than for T1 (HR, 0.37; 95% CI, 0.19-0.72; P = 0.003), whereas T2 did not differ from T1.

**Table 3:** Cox regression analysis of five pretransplant nutritional indices for overall mortality.

Variable	Units	Model 1		Model 2		Model 3	
		HR (95% CI)	P	HR (95% CI)	P	HR (95% CI)	P
CONUT	Per 1-SD increase	0.87 (0.70-1.08)	0.199	0.86 (0.69-1.07)	0.169	0.98 (0.74-1.29)	0.867
CONUT Group	Normal	Reference		Reference		Reference	
	Moderate malnutrition	1.14 (0.59-2.22)	0.690	1.11 (0.57-2.15)	0.767	1.62 (0.77-3.37)	0.201
	Severe malnutrition	0.66 (0.24-1.82)	0.421	0.63 (0.23-1.74)	0.372	1.40 (0.43-4.60)	0.581
GNRI	Per 1-SD increase	0.80 (0.64-0.98)	0.034	0.80 (0.64-0.98)	0.035	0.75 (0.58-0.98)	0.037
GNRI Group	High-risk level	Reference		Reference		Reference	
	Borderline to moderate risk	1.25 (0.64-2.43)	0.511	1.26 (0.65-2.46)	0.496	1.12 (0.54-2.35)	0.756
	Normal range	0.40 (0.17-0.97)	0.043	0.40 (0.17-0.97)	0.043	0.35 (0.13-0.95)	0.039
PNI	Per 1-SD increase	0.86 (0.70-1.06)	0.149	0.86 (0.70-1.06)	0.162	0.78 (0.61-1.01)	0.055
PNI Group	T1	Reference		Reference		Reference	
	T2	1.37 (0.83-2.25)	0.218	1.39 (0.84-2.28)	0.198	1.15 (0.67-2.00)	0.609
	T3	0.77 (0.44-1.35)	0.370	0.79 (0.45-1.38)	0.411	0.66 (0.35-1.26)	0.207
TCBI	Per 1-SD increase	1.02 (0.84-1.23)	0.849	1.02 (0.84-1.23)	0.871	1.05 (0.85-1.31)	0.639
TCBI Group	T1	Reference		Reference		Reference	
	T2	1.56 (0.88-2.76)	0.126	1.61 (0.91-2.85)	0.104	1.19 (0.66-2.15)	0.568
	T3	1.98 (1.15-3.42)	0.014	2.05 (1.18-3.56)	0.011	1.45 (0.80-2.64)	0.221
AGR	Per 1-SD increase	0.61 (0.47-0.78)	<0.001	0.61 (0.47-0.79)	<0.001	0.64 (0.48-0.84)	0.002
AGR Group	T1	Reference		Reference		Reference	
	T2	1.07 (0.67-1.71)	0.768	1.10 (0.69-1.76)	0.694	1.16 (0.70-1.90)	0.571
	T3	0.34 (0.18-0.63)	<0.001	0.34 (0.18-0.63)	<0.001	0.37 (0.19-0.72)	0.003

Model 1 was unadjusted. Model 2 adjusted for age and sex. Model 3 adjusted for age, sex, ln(AFP), maximum tumor diameter, tumor number, PVTT, and Child-Pugh class. Continuous HRs are per 1-SD increase. HR, hazard ratio; CI, confidence interval.

GNRI was inversely associated with mortality in all three continuous models; the Model 3 HR per 1-SD increase was 0.75 (95% CI, 0.58-0.98; P = 0.037). PNI had a borderline continuous association in Model 3 (HR, 0.78; 95% CI, 0.61-1.01; P = 0.055), without a consistent gradient across tertiles. The fully adjusted continuous estimates were null for CONUT (HR, 0.98; 95% CI, 0.74-1.29; P = 0.867) and TCBI (HR, 1.05; 95% CI, 0.85-1.31; P = 0.639). **Table 3** provides the grouped estimates for all adjustment levels.

### 3.3 Kaplan-Meier and restricted cubic spline analyses

**Figure 1:**
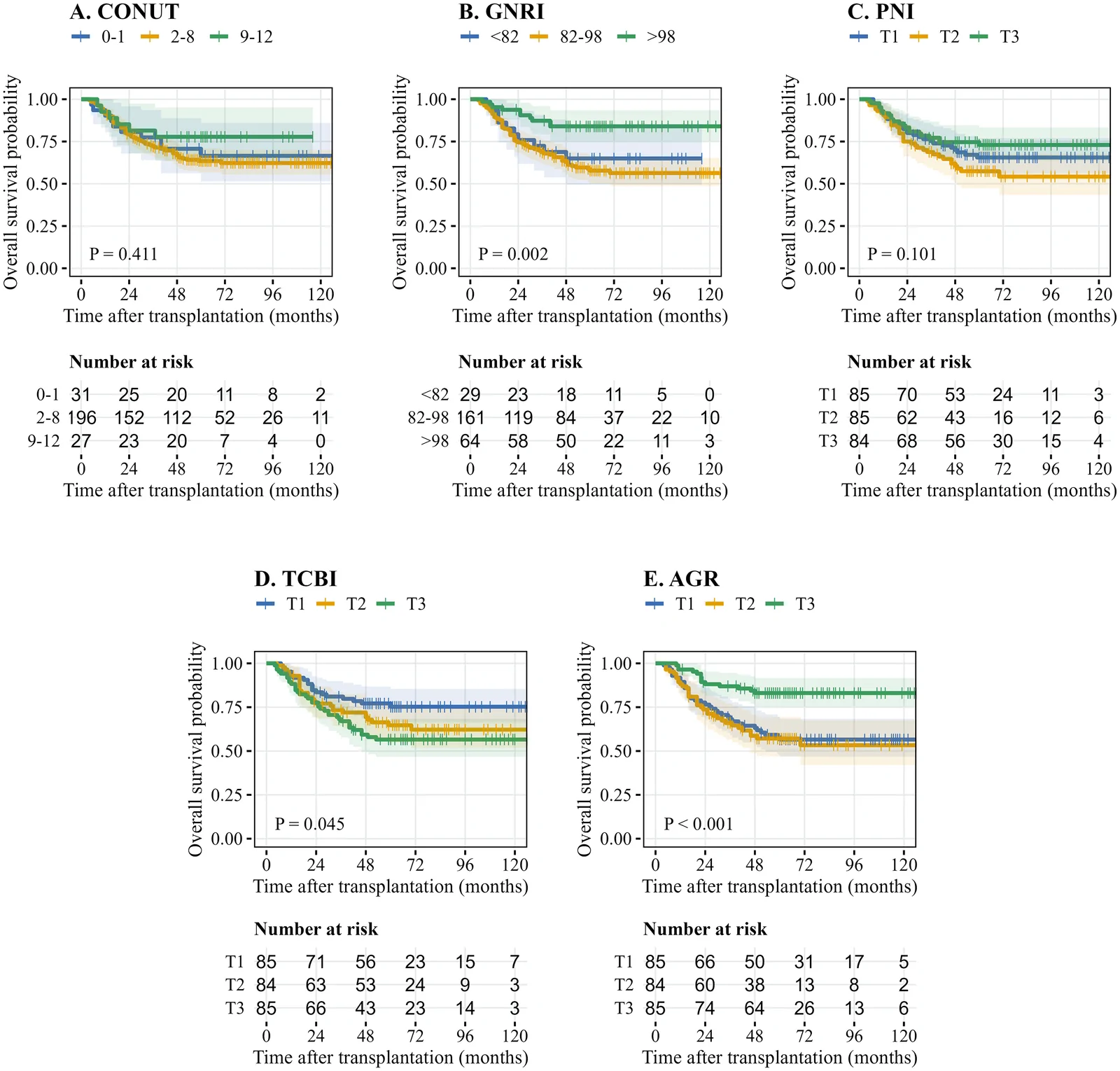
Kaplan-Meier estimates of OS according to five pretransplant nutritional indices: CONUT **(A)**, GNRI **(B)**, PNI **(C)**, TCBI **(D)**, and AGR **(E)**. Shading denotes 95% CIs; numbers at risk are shown below each panel. P values are from log-rank tests.

OS differed across GNRI categories (log-rank P = 0.002), TCBI tertiles (P = 0.045), and AGR tertiles (P < 0.001), but not across CONUT categories (P = 0.411) or PNI tertiles (P = 0.101) (**Figure 1**). For AGR, separation was greatest between T3 and the two lower tertiles; T1 and T2 remained close during much of follow-up.

**Figure 2:**
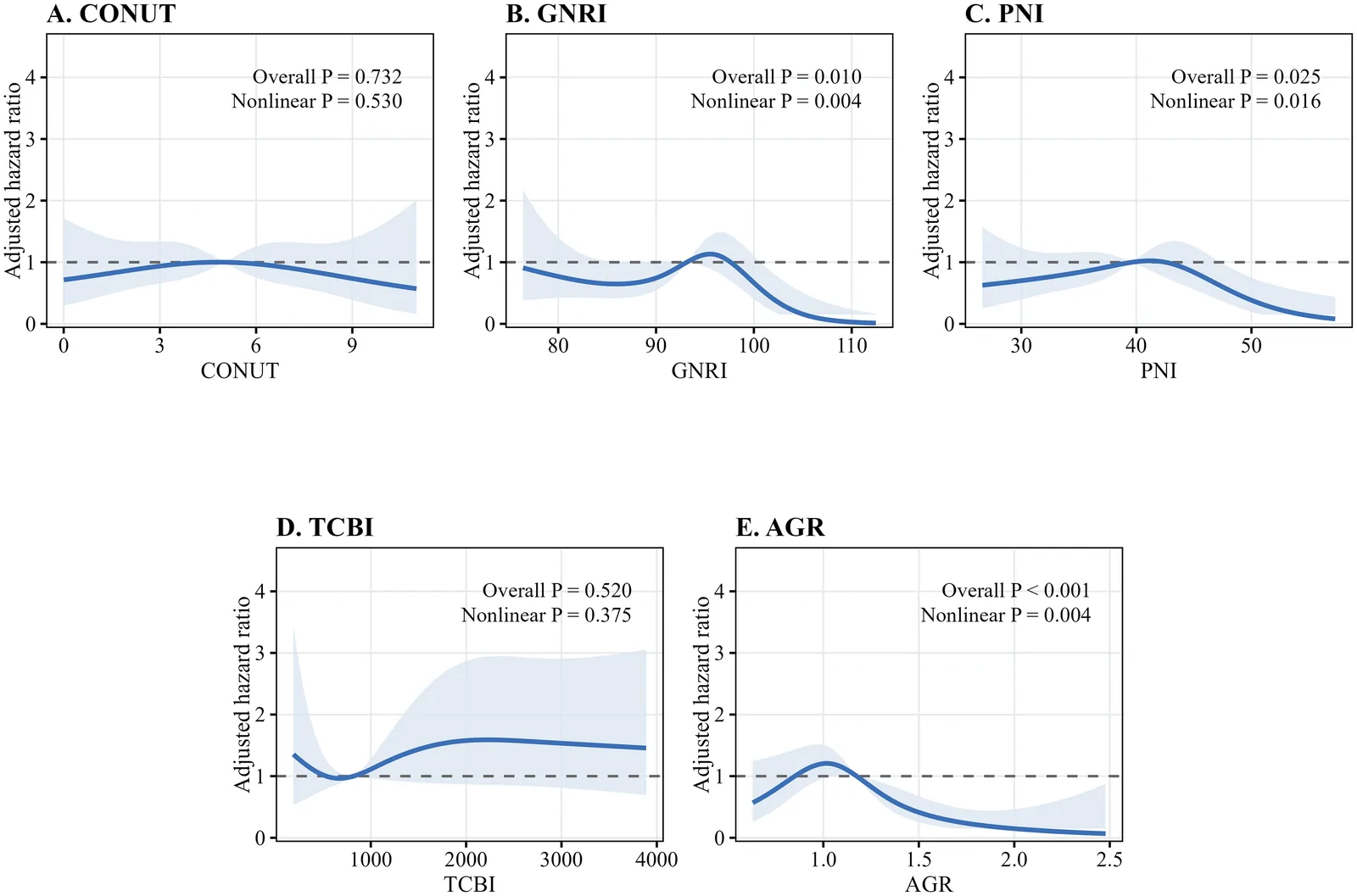
Adjusted restricted cubic spline associations of five pretransplant nutritional indices with overall mortality: CONUT **(A)**, GNRI **(B)**, PNI **(C)**, TCBI **(D)**, and AGR **(E)**. Lines show adjusted HRs and shading denotes 95% CIs. Models included age, sex, ln(AFP), maximum tumor diameter, tumor number, PVTT, and Child-Pugh class.

Restricted cubic spline results are shown in **Figure 2**. AGR was associated with mortality overall (P < 0.001), with evidence of nonlinearity (P = 0.004). GNRI also showed an overall association (P = 0.010) and nonlinearity (P = 0.004). For PNI, the corresponding P values were 0.025 and 0.016. No overall dose-response association was detected for CONUT or TCBI in the fully adjusted spline models.

### 3.4 AGR subgroup and Child-Pugh analyses

**Supplementary Figure 1** presents age-, BMI-, and Child-Pugh-stratified estimates for all five nutritional indices, while **Table 4** provides the broader AGR subgroup analysis. Adjusted AGR estimates were below 1.00 in most strata. The association was present among patients younger than 60 years (HR per 1-SD increase, 0.40; 95% CI, 0.25-0.63; P<0.001), men (HR, 0.60; 95% CI, 0.44-0.81; P<0.001), patients within or outside Milan criteria, patients without PVTT, patients with more than one tumor, and patients with tumors no larger than 5 cm. Several smaller strata had wide CIs. Interaction tests were nominally significant for age (P = 0.025) and PVTT (P = 0.020), but not for sex, BMI, Milan status, tumor number, or tumor size. These P values were not corrected for multiple testing.

**Table 4:** Association between AGR and overall mortality across subgroups.

Subgroup	Adjusted HR (95% CI)	P value	P for interaction
Overall	0.64 (0.48-0.84)	0.002	
Age, years			0.025
<60	0.40 (0.25-0.63)	<0.001	
>=60	0.91 (0.60-1.36)	0.634	
Sex			0.376
Female	0.97 (0.38-2.46)	0.941	
Male	0.60 (0.44-0.81)	<0.001	
BMI, kg/m2			0.611
<24	0.72 (0.44-1.18)	0.193	
>=24	0.55 (0.37-0.81)	0.003	
Child-Pugh class			0.070
A	0.38 (0.21-0.69)	0.002	
B/C	0.76 (0.56-1.02)	0.070	
Milan criteria			0.921
Outside	0.64 (0.46-0.90)	0.010	
Within	0.41 (0.20-0.83)	0.014	
Portal vein tumor thrombus			0.020
No	0.55 (0.40-0.76)	<0.001	
Yes	0.81 (0.39-1.66)	0.562	
Tumor number			0.642
1	0.75 (0.46-1.23)	0.256	
>1	0.58 (0.40-0.83)	0.003	
Maximum tumor diameter			0.951
<=5 cm	0.59 (0.41-0.86)	0.006	
>5 cm	0.76 (0.47-1.23)	0.264	

Adjusted HRs are per 1-SD increase in AGR. Models used the Model 3 adjustment set with the stratifying variable omitted. Interaction P values were obtained from nested models and were not corrected for multiple testing.

When Child-Pugh class was grouped as A versus B/C, the interaction did not reach conventional significance (P = 0.070). In the class-specific three-level analysis, the HR was 0.38 (95% CI, 0.21-0.69; P = 0.002) in class A, 0.65 (95% CI, 0.44-0.97; P = 0.034) in class B, and 1.31 (95% CI, 0.72-2.36; P = 0.374) in class C. The global AGR-by-Child-Pugh interaction was also nonsignificant (P = 0.058). Median AGR declined across worsening Child-Pugh classes, but precision was limited in class C (**Supplementary Table 1**).

### 3.5 Tumor recurrence and fine-gray analysis

**Figure 3:**
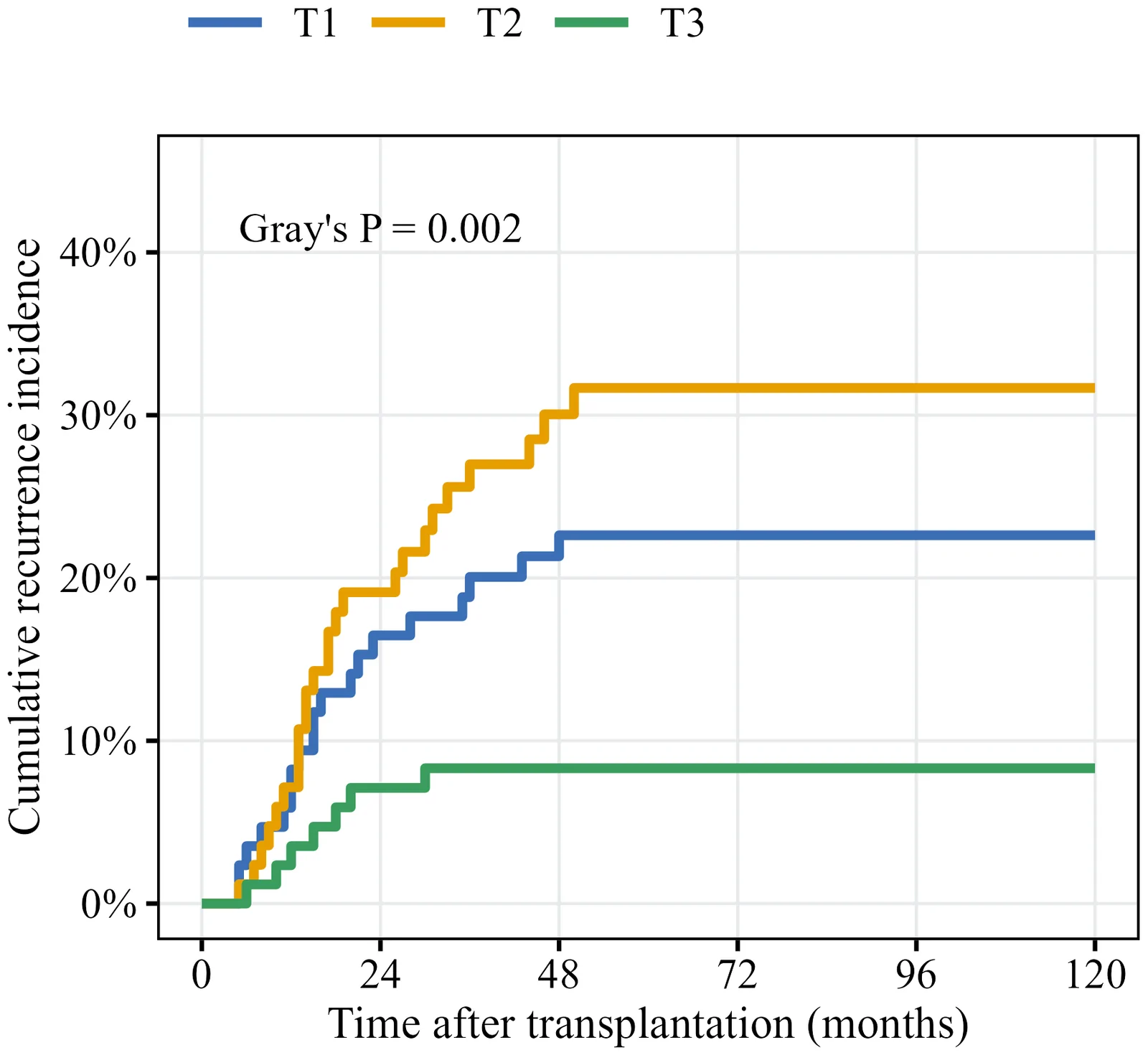
Cumulative incidence of HCC recurrence by AGR tertile, with death before recurrence treated as a competing event. The P value is from Gray’s test.

First recurrence occurred in 51 recipients. Forty died before any recorded recurrence and were treated as competing events; 163 remained alive and recurrence-free at censoring. Cumulative recurrence incidence differed across AGR tertiles (Gray’s P = 0.002; **Figure 3**), with the lowest incidence in T3.

Each 1-SD increase in AGR was associated with a lower recurrence subdistribution hazard in the unadjusted, age- and sex-adjusted, and fully adjusted Fine-Gray models. The Model 3 sHR was 0.66 (95% CI, 0.47-0.94; P = 0.022). In the categorical Model 3 analysis, the sHR was 0.38 for AGR T3 versus T1 (95% CI, 0.15-0.94; P = 0.035), while T2 did not differ from T1. Tumor number and PVTT also remained associated with recurrence. Full estimates are reported in **Table 5**.

**Table 5:** Fine-Gray regression analysis of AGR for time to recurrence.

Variable	Units	Model 1		Model 2		Model 3	
		sHR (95% CI)	P	sHR (95% CI)	P	sHR (95% CI)	P
AGR	Per 1-SD increase	0.65 (0.48-0.88)	0.006	0.65 (0.48-0.88)	0.006	0.66 (0.47-0.94)	0.022
AGR Group	T1	Reference		Reference		Reference	
	T2	1.42 (0.79-2.57)	0.243	1.50 (0.83-2.70)	0.179	1.46 (0.78-2.73)	0.242
	T3	0.34 (0.14-0.82)	0.017	0.35 (0.15-0.83)	0.017	0.38 (0.15-0.94)	0.035

Death before recurrence was treated as a competing event. Model 1 was unadjusted; Model 2 adjusted for age and sex; Model 3 additionally adjusted for ln(AFP), maximum tumor diameter, tumor number, PVTT, and Child-Pugh class. sHR, subdistribution hazard ratio.

### 3.6 Prediction-model comparisons

**Figure 4:**
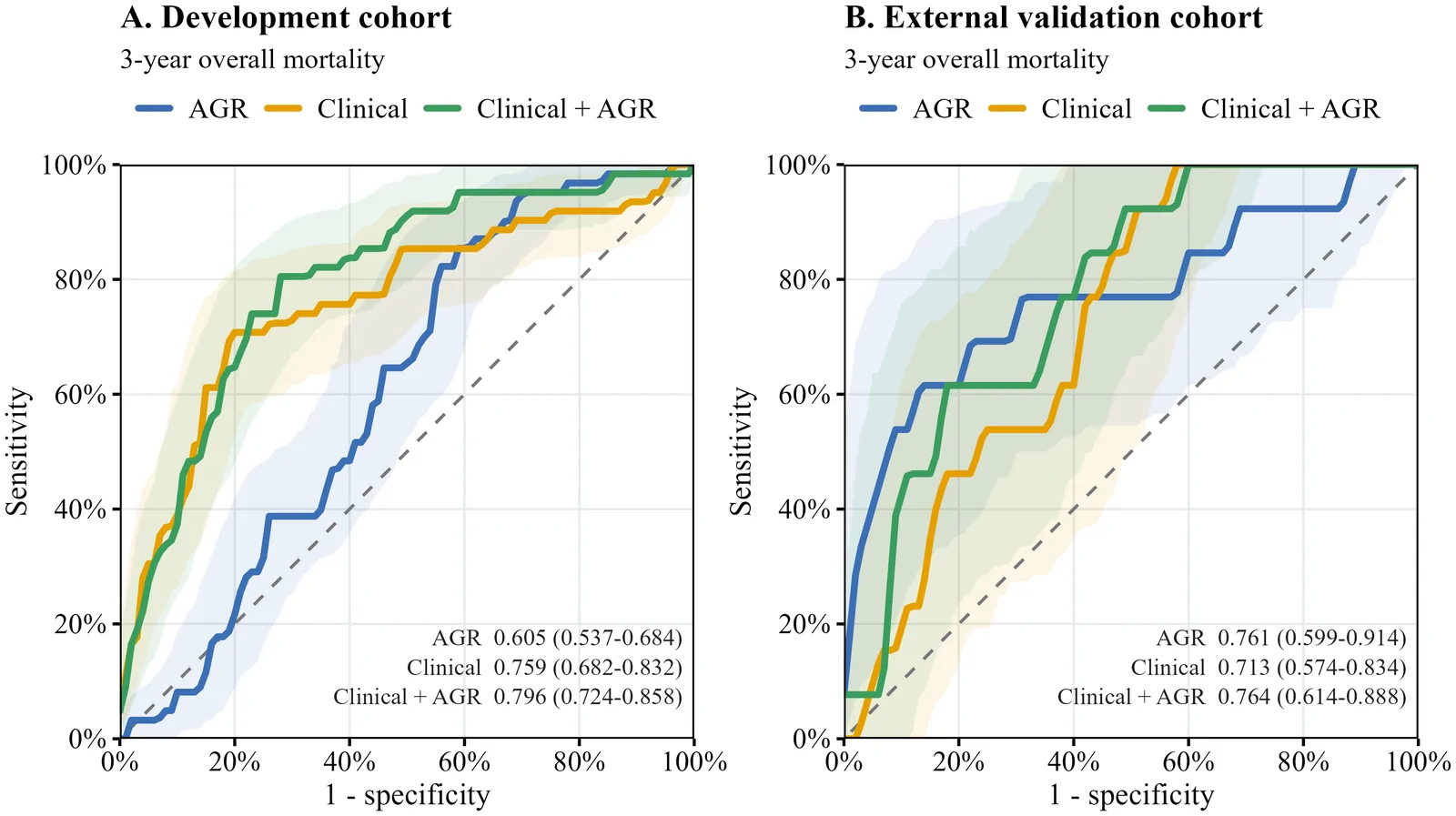
Time-dependent ROC analysis of 3-year overall mortality in the development cohort **(A)** and independent external validation cohort **(B)**. Curves compare AGR alone, the pretransplant clinical model, and the clinical model plus AGR. Shaded regions denote pointwise 95% bootstrap confidence intervals; inset values are AUCs with 95% bootstrap confidence intervals. Development-cohort curves show apparent full-sample discrimination, whereas external curves use fixed development-model coefficients without refitting.

In the common model-comparison sample, the C-index was 0.552 for BCLC, 0.658 for CNLC, 0.607 for AGR alone, 0.750 for the clinical model, and 0.780 for the clinical-plus-AGR model. At 3 years, the apparent time-dependent AUC was 0.605 (95% CI, 0.537-0.684) for AGR alone, 0.759 (95% CI, 0.682-0.832) for the clinical model, and 0.796 (95% CI, 0.724-0.858) for the clinical-plus-AGR model (**Figure 4**). Bootstrap-corrected AUCs for the clinical and clinical-plus-AGR models were 0.817 and 0.818 at 1 year, 0.723 and 0.760 at 3 years, and 0.758 and 0.787 at 5 years, respectively (**Supplementary Table 2**).

**Figure 5:**
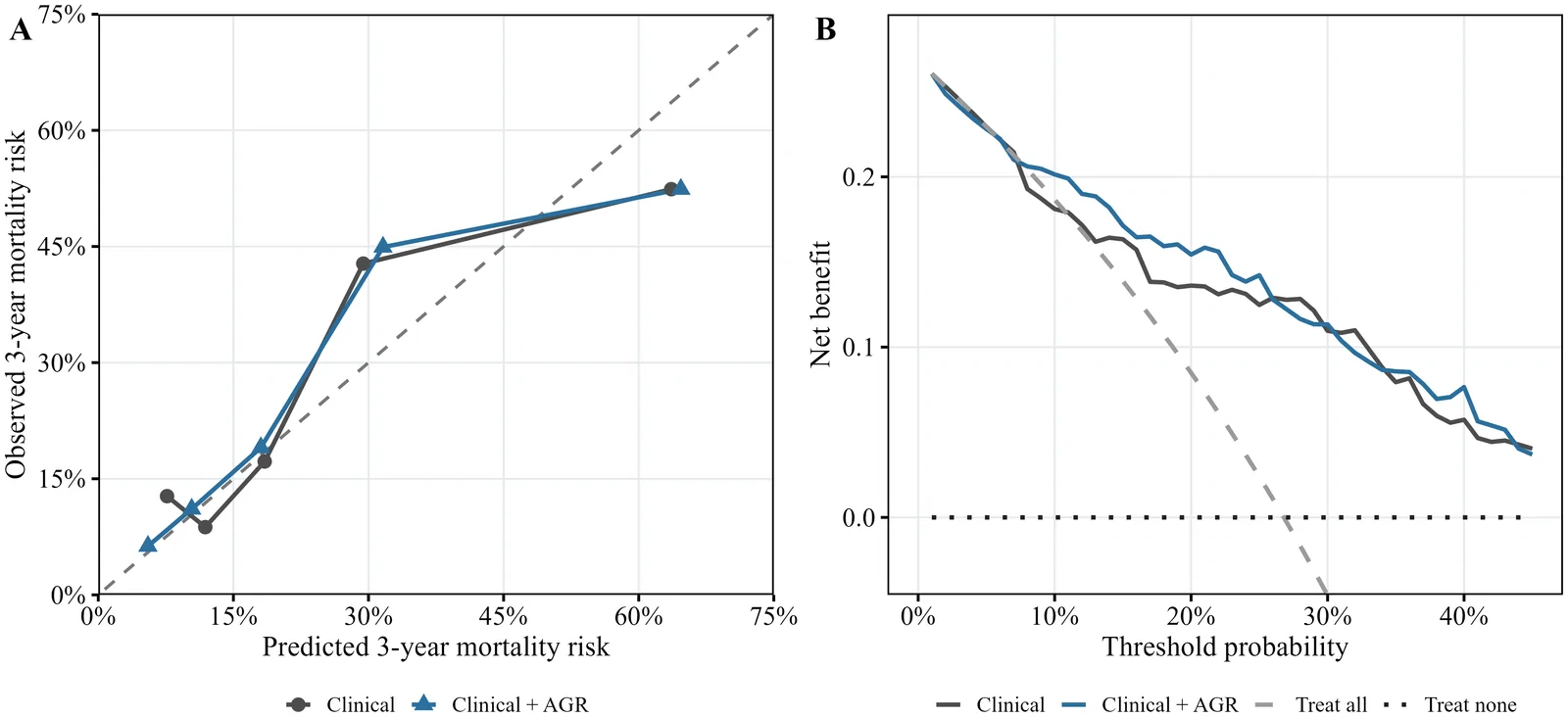
Internal assessment of OS models in the development cohort. **(A)** Bootstrap-corrected calibration at 3 years. **(B)** Decision curves at 3 years. The clinical models included age, sex, ln(AFP), maximum tumor diameter, tumor number, PVTT, and Child-Pugh class.

Three-year calibration showed broadly similar agreement for the clinical and clinical-plus-AGR models, with uncertainty at the higher predicted-risk range. Decision curves overlapped over part of the threshold range, with modest separation in selected intervals (**Figure 5**). The corresponding 1- and 5-year ROC curves are shown in **Supplementary Figures 3** and **4**. For TTR, bootstrap-corrected AUCs for the clinical and clinical-plus-AGR models were 0.607 and 0.604 at 1 year, 0.665 and 0.696 at 3 years, and 0.652 and 0.672 at 5 years. Brier scores changed little, indicating limited incremental recurrence discrimination (**Supplementary Table 2**).

### 3.7 Exploratory machine-learning analysis

**Figure 6:**
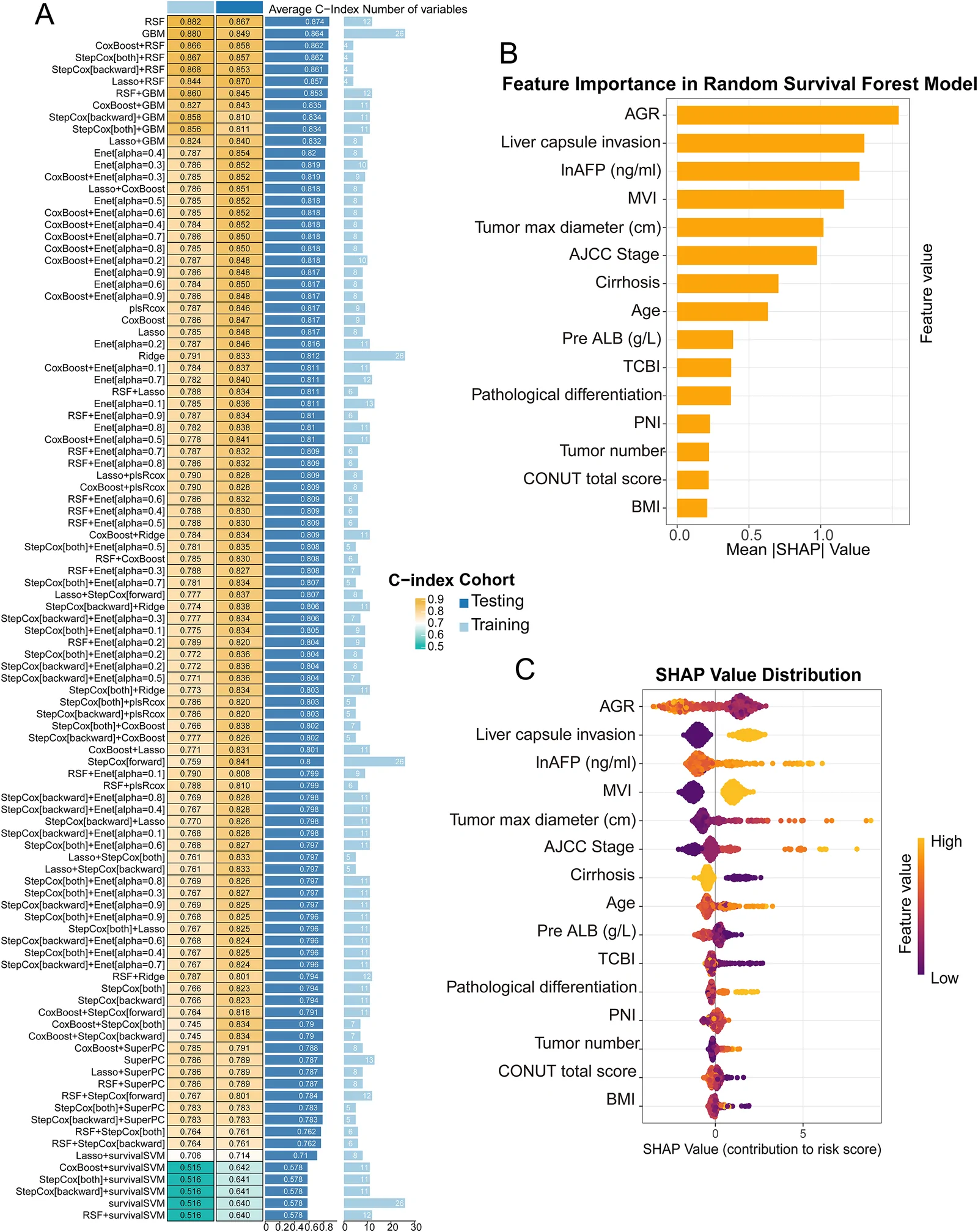
Exploratory machine-learning survival modelling and SHAP interpretation. **(A)** Performance comparison of candidate survival models based on training, testing, and mean C-index. **(B)** Feature-importance ranking in the selected random survival forest based on mean absolute SHAP values. **(C)** SHAP value distribution showing the contribution of individual variables to the model-derived risk score.

The random survival forest showed the highest overall discrimination among the exploratory survival-learning models, with a testing C-index of 0.882, a training C-index of 0.867, and a mean C-index of 0.874. Gradient boosting ranked second, with testing, training, and mean C-indices of 0.880, 0.849, and 0.864, respectively. AGR ranked first in the random-forest feature-importance and SHAP analyses, followed by capsule invasion, ln(AFP), MVI, maximum tumor diameter, and AJCC stage (**Figure 6**).

### 3.8 Immunosuppression sensitivity and external validation

The initial regimen was tacrolimus-based in 198 recipients (78.0%), cyclosporine-based in 51 (20.1%), and recorded as combined or switched TAC/CSA in five (2.0%). Sixty-six patients (26.0%) initially received MMF and five (2.0%) received an mTOR inhibitor. Cohort-wide occurrence data for infection within 90 days and acute rejection within 1 year were not available and were not treated as zero-frequency events. Adding initial CNI class to the fully adjusted OS model changed the AGR estimate from HR 0.64 (95% CI, 0.48-0.84; P = 0.002) to HR 0.66 (95% CI, 0.50-0.87; P = 0.004).

The independent external validation cohort included 58 recipients, 19 deaths, and 12 recurrences; median follow-up was 65.5 months. Compared with the development cohort, recipients in the external cohort were younger on average, had smaller and fewer tumors, were more often within Milan criteria, had no Child-Pugh C cases, and had a higher mean AGR. With development-model coefficients fixed, the C-index was 0.679 (95% CI, 0.544-0.814) for AGR alone, 0.645 (95% CI, 0.535-0.755) for the clinical model, and 0.686 (95% CI, 0.577-0.796) for the clinical-plus-AGR model. At 3 years, the corresponding AUCs were 0.761 (95% CI, 0.599-0.914), 0.713 (95% CI, 0.574-0.834), and 0.764 (95% CI, 0.614-0.888), respectively (**Figure 4**). At 1 year, the AUCs were 0.766, 0.631, and 0.699; at 5 years, they were 0.682, 0.680, and 0.718 (**Table 6**; **Supplementary Figures 3**, **4**). Calibration slopes for the clinical and clinical-plus-AGR models were 0.80 and 0.93, with wide confidence intervals; grouped calibration is shown in **Supplementary Figure 2**. Because only 12 recurrences occurred externally, TTR discrimination was treated as exploratory and was not used to rank models.

**Table 6:** External validation of fixed overall-survival models in the independent external validation cohort.

Model	C-index (95% CI)	1-year AUC (95% CI)	3-year AUC (95% CI)	5-year AUC (95% CI)	Calibration slope (95% CI)
AGR	0.679 (0.544-0.814)	0.766 (0.471-0.958)	0.761 (0.599-0.914)	0.682 (0.491-0.825)	3.25 (0.62-5.88)
Clinical	0.645 (0.535-0.755)	0.631 (0.449-0.821)	0.713 (0.574-0.834)	0.680 (0.529-0.807)	0.80 (0.02-1.59)
Clinical + AGR	0.686 (0.577-0.796)	0.699 (0.494-0.875)	0.764 (0.614-0.888)	0.718 (0.586-0.844)	0.93 (0.23-1.64)

The independent external validation cohort included 58 recipients and 19 deaths. Development-cohort coefficients, baseline hazards, standardization parameters, and AGR cut points were applied without refitting. At 1, 3, and 5 years, 52, 45, and 39 recipients remained at risk, respectively. AUC confidence intervals were obtained from 500 nonparametric bootstrap samples. AUC, area under the curve; CI, confidence interval.

## 4 Discussion

In this transplant cohort, AGR had the most consistent pattern among the five routine nutritional indices, but it was not the only marker associated with outcome. Higher AGR was related to lower overall mortality in the unadjusted, age- and sex-adjusted, and fully adjusted Cox models; the highest tertile also differed from the lowest. GNRI retained a smaller adjusted association, while PNI showed a nonlinear pattern and borderline continuous estimate. CONUT and TCBI were sensitive to scale or adjustment and did not retain independent continuous associations. The recurrence analysis led to a similar result for AGR after deaths without recurrence were treated as competing events. Adding AGR to the clinical OS model increased concordance and improved 3- and 5-year discrimination, although the 1-year AUC and several Brier-score estimates changed little. The random-survival-forest analysis provided complementary support, with AGR ranking ahead of the other candidate variables in that exploratory model. Taken together, these findings support AGR as a candidate adjunct to established tumor-related variables rather than a stand-alone prognostic instrument.

Recurrence after transplantation is clinically distinct from all-cause mortality because death can occur without recurrent HCC. Treating such deaths as ordinary censoring can overstate recurrence risk. We therefore used cumulative-incidence curves and Fine-Gray regression, with death before recurrence as a competing event. AGR retained an association with recurrence after adjustment for age, sex, AFP, tumor size, tumor number, PVTT, and Child-Pugh class. The estimate was consistent with the OS analysis, but its clinical interpretation is narrower: pretransplant AGR was associated with the subsequent incidence of documented recurrence in the presence of competing mortality.

AGR is readily calculated, but its biological meaning is broad. Albumin varies with hepatic synthesis, dietary intake, protein loss, fluid distribution, and systemic illness. Globulin comprises immunoglobulins and other protein fractions that can rise with chronic liver injury, inflammation, or infection. A low AGR may therefore arise through several pathways common to advanced liver disease and HCC (13, 21–24). This breadth may contribute to its reproducibility across clinical settings, while limiting mechanistic specificity. We did not measure cytokines, immunoglobulin subclasses, tumor-infiltrating lymphocytes, graft immune tolerance, or local immune remodeling. AGR should therefore be interpreted as a systemic biochemical correlate of recipient condition, not as a direct measure of antitumor immunity or immune surveillance.

Differences among the five indices can also be understood from their components. CONUT incorporates lymphocyte count and cholesterol in addition to albumin, yet lymphocyte counts were nearly identical between outcome groups and cholesterol was higher in the deceased group. PNI uses the same lymphocyte component and showed no orderly gradient across tertiles. GNRI retained an association, plausibly because of its albumin term, but body weight is difficult to interpret in recipients with edema or ascites. TCBI is dominated by lipids and body weight and was attenuated after adjustment for tumor and liver characteristics. These observations do not make one construct biologically superior; they show that indices developed in different populations are not interchangeable (9–14, 21). Continuous modeling and explicit assessment of dose-response shape are preferable to choosing a favorable data-derived threshold.

The Child-Pugh findings require caution. Median AGR declined from class A to class C, as expected because albumin contributes to Child-Pugh classification and advanced liver dysfunction can alter globulin concentrations. AGR was associated with mortality within classes A and B but not class C; however, neither the A-versus-B/C interaction nor the global three-level interaction reached conventional significance. The class C estimate was imprecise. Overlap with hepatic reserve, a narrower AGR distribution in advanced disease, small subgroup size, and competing causes of death are all plausible explanations. The nominal age and PVTT interactions likewise arose from multiple exploratory comparisons and require independent confirmation. AGR should be interpreted alongside Child-Pugh class, tumor burden, AFP, vascular invasion, and transplant selection criteria.

A pretransplant measurement cannot represent the subsequent course of immunosuppression or post-transplant complications. Exposure to CNIs, MMF, and mTOR inhibitors changes with renal function, rejection, infection, and recurrence risk, and immunosuppressive intensity itself may influence HCC recurrence (8, 25). Baseline AGR was measured before these events, but later treatment and complications may modify its association with outcome. Initial regimen and CNI class were available; additional CNI adjustment changed the AGR estimate only slightly. By contrast, cohort-wide infection and acute-rejection incidence, drug levels, regimen conversion, rejection severity, and serial laboratory values were unavailable. Cause-of-death classifications were not used as substitutes for complication incidence. Longitudinal studies with time-updated treatment and biochemical measurements will be required to separate baseline prognosis from post-transplant evolution.

The prediction analyses place the biomarker findings in a more clinically relevant setting. BCLC and CNLC alone had lower concordance than the multivariable clinical model, and adding AGR produced a modest increase in OS discrimination, most clearly at 3 and 5 years. AGR alone discriminated poorly in the development cohort but performed similarly to the multivariable models at 3 years in the external cohort. That contrast, together with the broad external confidence intervals, argues against treating AGR as a stand-alone prediction tool. The transported clinical-plus-AGR model nevertheless had numerically higher AUCs than the clinical model at all three horizons. Calibration and decision-curve separation remained limited, and the external cohort differed in tumor burden, hepatic reserve, Milan status, and AGR distribution. These findings support further validation of AGR as a complementary variable within a clinical model, not as a substitute for established tumor-based assessment (26–35).

This study has several limitations. Its retrospective design permits measurement variation, residual confounding, and outcome misclassification. Early infection and acute-rejection incidence were not available across the development cohort and could not be analyzed as longitudinal exposures. The independent external validation cohort was small, with 58 recipients, 19 deaths, and 12 recurrences; its case mix differed from that of the development cohort, confidence intervals were wide, and external TTR analyses were secondary. The tertile cut points are cohort-dependent and should not be regarded as universal thresholds. Event counts limited the complexity of interaction and competing-risk models. Multiple indices and secondary analyses were examined without multiplicity correction, and no molecular, histological, serial immunosuppressive, or longitudinal nutritional data were available. Larger prospective multicenter studies should prespecify recurrence surveillance, treatment collection, model parameters, and thresholds.

## 5 Conclusion

Among five indices derived from routine pretransplant measurements, AGR showed the most consistent associations with OS and competing-risk recurrence after liver transplantation for HCC. Adding AGR produced modest, time-dependent gains in the development cohort, with changes in the same direction in a small independent external validation cohort. Tumor burden, AFP, and vascular invasion remained central to prognosis. AGR should be considered a complementary candidate marker pending larger prospective multicenter validation.

## Data Availability

The participant-level datasets generated and analyzed in this study are not publicly available because they contain clinical information subject to institutional ethics and privacy restrictions. Requests to access the datasets should be directed to the corresponding authors and will be considered only where permitted by the relevant ethics committees and participating institutions.
