# Supplementary material for: Pretransplant albumin-to-globulin ratio and long-term outcomes after liver transplantation for hepatocellular carcinoma: a two-center cohort study
Source: Front Oncol. 2026 Aug 24;16:1885468. doi: 10.3389/fonc.2026.1885468 (PMC13547057; doi:10.3389/fonc.2026.1885468)
Supplement: Supplementary file 1 [file DataSheet1.docx]

**Supplementary Material**

**Supplementary methods**

The development cohort comprised 254 unique recipients, and the independent external validation cohort comprised 58 adults with HCC who underwent liver transplantation in 2018 at First Central Hospital of Tianjin Medical University. Eligibility criteria, variable definitions, and outcome coding were harmonized. Development-model coefficients, baseline hazards, standardization parameters, and cut points were applied externally without refitting or recalibration; missing model covariates were not imputed.

Cohort-wide data on infection within 90 days and acute rejection within 1 year were not reliably available in the development cohort, and missing records were not interpreted as absence. Initial immunosuppression was summarized descriptively, and initial CNI class was added to the fully adjusted OS model in a sensitivity analysis.

Cumulative/dynamic ROC curves compared AGR alone, the pretransplant clinical model, and the clinical-plus-AGR model for 1-, 3-, and 5-year OS. Pointwise confidence bands and AUC confidence intervals were estimated from 500 nonparametric bootstrap samples within each cohort. The 3-year curves are shown in the main manuscript; the 1- and 5-year curves are shown in Supplementary Figures S3 and S4.

**Supplementary results**

The external validation cohort included 58 recipients, 19 deaths, and 12 recurrences, with a median follow-up of 65.5 months. Recipients were younger, had lower tumor burden, were more often within Milan criteria, and included no Child-Pugh class C cases. These differences were treated as between-centre case-mix variation; all development-model parameters were applied unchanged without refitting or recalibration.

Initial CNI therapy was tacrolimus based in 198 recipients (78.0%), cyclosporine based in 51 (20.1%), and recorded as combined or switched in five (2.0%). Adding initial CNI class to the fully adjusted OS model changed the AGR estimate from HR 0.64 (95% CI, 0.48-0.84; P=0.002) to HR 0.66 (95% CI, 0.50-0.87; P=0.004).

| Table S1. AGR distribution and adjusted association with overall mortality by Child-Pugh class | | | |
| --- | --- | --- | --- |
| **Child-Pugh class** | **AGR, median (IQR)** | **Adjusted HR (95% CI)** | **P** |
| A | 1.344 (1.087-1.645) | 0.38 (0.21-0.69) | 0.002 |
| B | 1.190 (1.012-1.462) | 0.65 (0.44-0.97) | 0.034 |
| C | 0.978 (0.787-1.246) | 1.31 (0.72-2.36) | 0.374 |

Median AGR decreased from Child-Pugh class A to class C. The adjusted association was evident in classes A and B, whereas the class C estimate had a wide confidence interval. The global three-level AGR-by-Child-Pugh interaction was not statistically significant (P=0.058), and the analysis was interpreted as exploratory.

| Table S2. Discrimination of prespecified models in the development cohort | | | | |
| --- | --- | --- | --- | --- |
| **Model** | **C-index** | **1-year AUC** | **3-year AUC** | **5-year AUC** |
| **A. Overall survival** | | | | |
| BCLC | 0.552 | 0.530 | 0.522 | 0.553 |
| CNLC | 0.658 | 0.679 | 0.634 | 0.656 |
| AGR | 0.607 | 0.578 | 0.615 | 0.594 |
| Clinical | 0.750 | 0.817 | 0.723 | 0.758 |
| Clinical + AGR | 0.780 | 0.818 | 0.760 | 0.787 |
| **B. Time to recurrence** | | | | |
| Milan criteria | 0.648 | 0.610 | 0.662 | 0.671 |
| Clinical | 0.737 | 0.607 | 0.665 | 0.652 |
| Clinical + AGR | 0.756 | 0.604 | 0.696 | 0.672 |

For OS, the C-index is Harrell's concordance; for TTR, 3-year time-dependent concordance is reported. The 1-, 3-, and 5-year AUCs are optimism-corrected estimates from 200 bootstrap resamples. External OS performance is reported in Table 6 of the main manuscript. AUC, area under the curve; OS, overall survival; TTR, time to recurrence.

**Supplementary figures**

Figure S1. Exploratory subgroup associations of CONUT (A), GNRI (B), PNI (C), TCBI (D), and AGR (E) with overall mortality. Squares show adjusted HRs per 1-SD increase and horizontal lines denote 95% CIs. Interaction P values are nominal.

AGR estimates were below 1.00 in most examined strata, but several subgroup confidence intervals were wide. Interaction findings were not adjusted for multiplicity and were not used to redefine the primary model.

Figure S2. Grouped calibration of fixed overall-survival models in the independent external validation cohort at 3 and 5 years. Points denote observed mortality within grouped predictions; the dashed diagonal denotes perfect calibration.

Grouped estimates were broadly compatible with the ideal line, but uncertainty increased at higher predicted risks. The small number of external deaths limits detailed assessment of calibration shape.

Figure S3. Time-dependent ROC analysis of 1-year overall mortality in the development and independent external validation cohorts. Curves compare AGR alone, the pretransplant clinical model, and the clinical model plus AGR. Shading denotes pointwise 95% bootstrap confidence intervals; inset values are AUCs with 95% bootstrap confidence intervals.

The development clinical models had similar 1-year discrimination. External estimates were less precise, and the wide intervals do not support ranking the three models at this early horizon.

Figure S4. Time-dependent ROC analysis of 5-year overall mortality in the development and independent external validation cohorts. Curves compare AGR alone, the pretransplant clinical model, and the clinical model plus AGR. Shading denotes pointwise 95% bootstrap confidence intervals; inset values are AUCs with 95% bootstrap confidence intervals.

At 5 years, the clinical-plus-AGR model had the highest point estimate in both cohorts, although confidence intervals overlapped, particularly in external validation.
